# The Application of Nucleic Acid Probe–Based Fluorescent Sensing and Imaging in Cancer Diagnosis and Therapy

**DOI:** 10.3389/fchem.2021.705458

**Published:** 2021-06-01

**Authors:** Ge Huang, Chen Su, Lijuan Wang, Yanxia Fei, Jinfeng Yang

**Affiliations:** ^1^ Department of Medicine, University of South China, Hengyang, China; ^2^ Department of Anesthesiology and Pain Medicine, Hunan Cancer Hospital/The Affiliated Cancer Hospital of Xiangya School of Medicine, Changsha, China

**Keywords:** nucleic acid probes, fluorescent sensing, fluorescent imaging, cancer diagnosis, cancer therapy

## Abstract

It is well known that cancer incidence and death rates have been growing, but the development of cancer theranostics and therapeutics has been a challenging work. Recently, nucleic acid probe–based fluorescent sensing and imaging have achieved remarkable improvements in a variety of cancer management techniques, credited to their high sensitivity, good tolerance to interference, fast detection, and high versatility. Herein, nucleic acid probe–based fluorescent sensing and imaging are labeled with advanced fluorophores, which are essential for fast and sensitive detection of aberrant nucleic acids and other cancer-relevant molecules, consequently performing cancer early diagnosis and targeted treatment. In this review, we introduce the characteristics of nucleic acid probes, summarize the development of nucleic acid probe–based fluorescent sensing and imaging, and prominently elaborate their applications in cancer diagnosis and treatment. In discussion, some challenges and perspectives are elaborated in the field of nucleic acid probe–based fluorescent sensing and imaging.

## Introduction

Nucleic acids (DNA and RNA) are one of the most essential components for organisms. Nucleic acid mutations, such as DNA translocations (Javadekar and Raghavan, 2015), small insertions and deletions (indels), and single-nucleotide polymorphisms (SNPs) (Mohlendick et al., 2019), are frequent events during cancer progression. Rapid progress in fluorescence-based nucleic acid probes is beneficial for studying the structural and conformational polymorphisms of nucleic acids and further investigating their variability, internal dynamics, and interactions with proteins, metabolites, and targeting drugs at the sub-molecular level (Sinkeldam et al., 2010; Michel et al., 2020).

Specific nucleic acid probes hold with a particular sequence, and they can recognize a broad range of targets, such as metal ions, small organic molecules, proteins, and even viruses or cells (Xiang and Lu, 2011; Huang et al., 2019; Bai et al., 2020). Nucleic acid probes mainly include DNA and RNA probes. DNA probes are useful tools for elaborating the biological processes of nucleic acid amplification, ligation, duplication, and transcription (Wu et al., 2014; Jia et al., 2015). SNPs are the most common DNA variations, and the multi-color SNP probes can discriminate four SNP variants with unique fluorescence colors, but the ideal multi-color SNP probes remain to be explored (Obliosca et al., 2013). RNA probes are responsible for severe DNA interference due to the similar structures between DNA and RNA, which restrains the exploration of RNA probes (Wang et al., 2016; Yao et al., 2018).

Generally, the specific structures of nucleic acid probes are beneficial to fabricate molecular computing devices, nanobiotechnology, and biomedical technology (Pu et al, 2014), like nucleic acid probe–based fluorescent sensing and imaging platforms. Fluorophores are crucial for the improvement of nucleic acid probe–based fluorescent sensing and imaging (Ebrahimi et al., 2014). Organic or conventional fluorophores have low quantum yield and poor photostability (Xu et al., 2018). Importantly, an increasing number of novel fluorescent nanomaterials have been developed, such as quantum dots (QDs), silver nanoclusters (AgNCs), gold nanoparticles (AuNPs), upconversion nanomaterials, and cationic conjugated polymers (CCPs) (Xu et al., 2016; Borghei et al., 2019). These fluorophores are characterized with better brightness, photostability, and size-tunable fluorescence spectrum and can directly or indirectly recognize specific targets with different patterns, such as hydrogen bonds, single-stranded DNA (ssDNA)/RNA hybridization, aptamer–target binding, enzyme inhibition, and enzyme-mimicking activity (Adinolfi et al., 2017).

To date, cancer mortality has been increasing around the word, chemotherapy and radiotherapy are widely used in cancer clinical treatment, but long-term treatment with chemotherapy drugs and radiotherapy can lead to multi-drug resistance, bone marrow suppression, and other adverse reactions (Kovacs et al., 2018; Zhou et al., 2018). Therefore, the development of effective treatment and early diagnosis becomes the key to decrease the death rate (He et al., 2019; Zhao et al., 2019). Since nucleic acid probe–based fluorescent sensing and imaging are attractive ways to identify the status of disease development, they have been widely investigated for usage in the early diagnosis and targeted treatment of various cancers by transforming biorecognition events into an amplified fluorescence signal (Mo et al., 2017; Lou et al., 2019; Li et al., 2021). Such tools allow for direct molecular recognition between nucleic acid probes and tested targets in living cells and tissues, which can quantitatively discriminate the amount and position of mutated DNA by producing an easily recordable and interpretable fluorescence signal (Hamd-Ghadareh et al., 2017).

Taken together, nucleic acid probe–based fluorescent sensing and imaging platforms are of great benefit to detect the positions and concentrations of cancer-relevant targets (Gao et al., 2018). Thereby, we would comprehensively elaborate the application of nucleic acid probe–based fluorescent sensing and imaging platforms in cancer diagnosis and therapy. In order to facilitate the development of innovative nucleic acid probe–based fluorescent sensing and imaging systems, some challenges and perspectives would be discussed.

## The Application of Nucleic Acid Probe–Based Fluorescent Sensing in Cancer Diagnosis and Treatment

**FIGURE 1:**
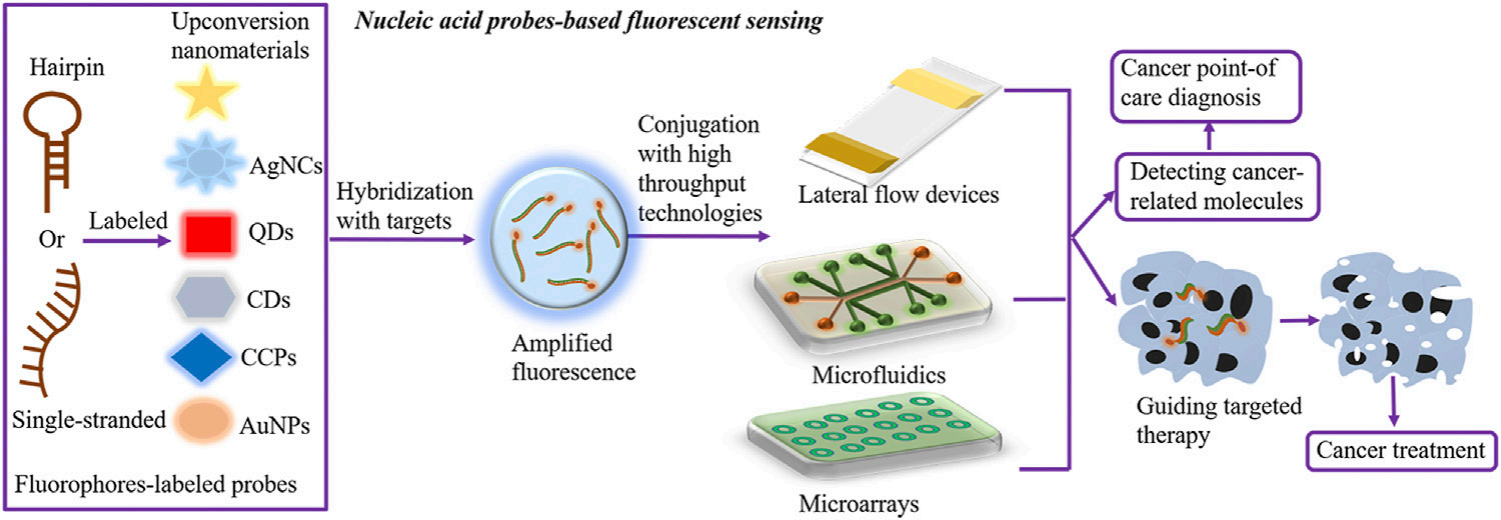
Nucleic acid probe–based fluorescent sensing. Hairpin or single-stranded nucleic acid probes are labeled with fluorophores to construct nucleic acid probe–based fluorescent sensing platforms. Effective fluorophores comprise QDs, CDs, AgNCs, AuNPs, CCPs, and upconversion nanomaterials. In the presence of targets, fluorophore-labeled probes hybridize targets and transmit the amplified fluorescence signal, further detecting the levels of cancer-relevant molecules and facilitating oncogene-guided individual therapy. In addition, the conjugation of fluorescent sensing with high-throughput microdevices, such as lateral flow devices, microfluidics, and microarrays, has shown distinguished advantages in cancer point-of-care diagnosis. AgNCs, silver nanoclusters; AuNPs, gold nanoparticles; CDs, carbon dots; QDs, quantum dots; CCPs, cationic conjugated polymers.

There are numerous outstanding fluorophores utilized for nucleic acid probe–based sensing, comprising QDs, carbon dots (CDs), AgNCs, AuNPs, CCPs, and upconversion nanomaterials. Presently, the fluorescence intensity-based measurement is extensively used, in which the fluorescence intensity varies based on the levels of targets, leading to the accurate and quantitative measurement of cancer-relevant molecules. Prospectively, conjugation of nucleic acid probe–based fluorescent sensing with high-throughput microdevices, such as lateral flow devices, microfluidics, and microarrays, has shown distinguished advantages in cancer point-of-care diagnosis and oncogene-guided individual therapy (Figure 1).

### Nucleic Acid Probe–Based Fluorescent Sensing Platforms in Cancer Diagnosis

Fluorescence biosensors are valuable tools for early diagnosing of cancer with precise and *in situ* monitoring of the spatiotemporal changes of miRNAs or proteins and identifying DNA mutations, such as single labeled molecular beacons (MBs) (FAM-MBs, with carboxyfluorescein and without quencher), a label-free beacon (AIE-MBs, without fluorogen and quencher), enzyme/nanomaterial-free and dual amplification, peptide nucleic acid (PNA), flow cytometry (FCM), nucleic acid aptamers–CDs, and enzymatic reaction–modified fluorescence sensing (Table 1).

**TABLE 1:** Application of nucleic acid probe–based fluorescent sensing in cancer diagnosis and treatment.

Fluorogenic biosensing	Probes	Cancers	Targets	Application	Ref.
FAM-MBs/AIE-MBs	ssDNA	Bladder cancer	Telomerase	Diagnosis	Ou et al. (2017)
An acceptor fluorophore dye	ssDNA	Breast cancer	miRNA let-7a	Diagnosis	Qiao et al. (2020)
An enzyme/nanomaterial-free and dual amplification	ssDNA	Various cancers	miRNA-141	Diagnosis	Wei et al. (2016)
PNA probes	ssDNA	Prostate cancer	miRNA-141 and miRNA-375	Diagnosis	Metcalf et al. (2016)
FCM-based DNA probes	ssDNA	Breast cancer	miRNA-21 and miRNA-141	Diagnosis	Peng et al. (2019a)
Nucleic acid aptamers–CDs	ssDNA	Various cancers	Cyt c	Diagnosis	Ghayyem and Faridbod (2018)
MP-MBs	ssDNA	Various cancers	p53	Treatment	Xu et al. (2015)
THP-RCA-MBs	ssDNA	Various cancers	STAT3	Treatment	Song et al. (2019)
IB-RCA-MBs	ssDNA	CRC	Kras gene codon 12	Treatment	Li et al. (2016)
PMMA-NPs	ssDNA	Lung cancer	Survivin mRNA	Treatment	Adinolfi et al. (2017)

AgNCs, silver nanoclusters; AIE-MBs, a label-free beacon; AuNPs, gold nanoparticles; CRC, colorectal cancer; Cyt c, cytochrome c; FCM, flow cytometry; IB-RCA, increasingly branched rolling circle amplification; MBs, molecular beacons; MP, multifunctional primer; PMMA-NPs, polymethylmethacrylate nanoparticles; PNA, peptide nucleic acid; ssDNA, single-stranded DNA; THP-RCA, ultrasensitive rolling circle amplification.

Graphene oxide (GO) is a typical nanomaterial that holds exceptional optical, electrical, mechanical, and chemical properties (Lin et al., 2014). It has attracted enormous attention in the study of DNA-based sensors by interacting with ssDNA through π–π stacking interactions (Tang et al., 2015). Human telomerase has been considered a promising cancer marker. The single labeled FAM-MBs are designed to detect telomerase activity with the aid of GO. To further simplify this structure, the more sensitive label-free AIE-MBs are constructed to monitor telomerase activity based on the enhanced fluorescence production (Ou et al., 2017). But the label-free AIE-MBs could carry a high signal-to-background ratio. Presently, their applications in bladder cancer diagnosis have been reported (Ou et al., 2017).

MicroRNAs (miRNAs) serve as ponderable serum cancer biomarkers due to their functions in modulating oncogenic pathways (Ndzi et al., 2019). Numerous nucleic acid probe–based fluorescent sensors have a great value in evaluating the serum concentrations of miRNAs (Yamamura et al., 2012). For instance, there are two programmable DNA probes labeled with either a donor or an acceptor fluorophore dye. In the presence of targets, the fluorescent sensing platform contributes to fluorescence resonance energy transfer (FRET) and signal amplification with a cascade hybridization reaction. The assay can sensitively detect the concentration of miRNA let-7a at single-cell resolution and discriminate let-7a from other highly homologous miRNAs in different molecular subtypes of breast cancer (Qiao et al., 2020). Nevertheless, the target-triggered and self-assembly character offers a high signal-to-noise ratio and strong read-out ratio, subsequently allowing for an effective detection at low abundance.

miRNA-141 is important for accelerating epithelial to mesenchymal transition (EMT) (Bhardwaj et al., 2017). An enzyme/nanomaterial-free and dual amplified strategy is developed for highly sensitive detection of miRNA-141 by combining hybridization chain reaction (HCR) and catalytic hairpin assembly (CHA) amplification (Wei et al., 2016). The HCR and CHA synergistically generate a remarkably amplified fluorescence signal, and the fluorescence signal intensity represents the concentration of the miRNA-141 target. Meanwhile, the platform can differentiate miRNA-141 from its family members and be expanded for designing DNA hairpin probes (Wei et al., 2016).

In addition, a novel PNA probe–based fluorogenic biosensor is designed to selectively target the miRNA-141 biomarker in serum without amplification step. In this system, PNAs are engineered with uncharged oligonucleotide analogs. And PNAs are capable of hybridizing to complementary targets with high affinity and specificity, further analyzing the concentrations of circulating miRNA-141 and miRNA-375, which have been applied for sensitively diagnosing prostate cancer (Pca) (Metcalf et al., 2016). In addition, fluorophore-labeled PNA probes are able to quantitatively and specifically detect multiplexed miRNAs in living cancer cells when conjugated with the nano metal–organic framework (NMOF) vehicle, and the release of PNAs from the NMOF would lead to the recovery of fluorescence (Wu et al., 2015). Innovatively, the interaction between immobilized PNA probes and DNA targets leads to enzyme-catalyzed pigmentation, allowing for simple visual read-out with up to 100% accuracy (Jirakittiwut et al., 2020).

Furthermore, a simpler DNA probe sensor has been creatively presented through integrating with FCM, which is based on the double key “unlocked mechanism” and the fluorescence enrichment signal amplification (Peng et al., 2019a; Oldham et al., 2020). In the sensor, fluorescent particle (FS)–labeled hairpin DNA probes (HDs) serve as the lock of “unlocked mechanism” and specifically hybridize with the probes on polystyrene (PS) microparticles. In the presence of miRNA targets, both miRNA targets and duplex-specific nuclease (DSN) act as the double key to specifically unlock HDs and increase the enrichment of HDs on PS microparticles. Then, the unlocked fluorescent probes lead to the enrichment of the fluorescent signal (Peng et al., 2019b). The FCM-based DNA probe sensor is allowed to measure miRNA-21 and miRNA-141 in breast cancer blood samples with higher sensitivity (Peng et al., 2019a). The whole procedure does not need a complex purification process, indicating a simplified FCM-based nucleic acid probe fluorescent sensing platform.

Protein also exerts enormous functions in diverse pathological activities, which provides effective targets for cancer diagnosis. For example, cytochrome c (Cyt c), a heme protein, is a significant biomarker for apoptosis (Chen et al., 2021). Cyt c–specific nucleic acid aptamers have the strong binding affinity to Cyt c. The detection of Cyt c relies on the interaction of nucleic acid aptamers with fluorescent CDs. In the presence of Cyt c, the interaction between nucleic acid aptamers and Cyt c would result in the release of CDs and fluorescence production, and the intensity of fluorescence is proportional to the concentration of Cyt c (Ghayyem and Faridbod, 2018). Therefore, the nucleic acid aptamer–CD sensing platform could be used for detecting various cancer-related proteins through designing target-specific nucleic acid aptamers, but CDs can only adsorb ssDNA probes via π–π interaction.

Nucleic acids serve as substrates of nucleic acid enzymes, and enzymatic reaction–mediated fluorescence sensing is a versatile avenue to improve the sensitivity of cancer diagnosis when combining with target-dependent cycling amplification (Allinson, 2010). In detail, when probe strands recognize the target DNA strands, the probe–target complexes are instantly digested by a specific enzyme to emit a fluorescence signal. Then, the released target DNA strands immediately react with another probe and give out a stronger signal (Zuo et al., 2010). Exonuclease III (Exo III) is one of the DNA-repair enzymes Chen et al. (2019a), and it is inclined to be recognized by MBs. MB-labeled fluorescence probes can effectively cleave Exo III and stimulate DNA-dependent signal recycling amplification, further testifying DNA mutation and diagnosing cancer (Zuo et al., 2010; Chen et al., 2019b).

In this work, a novel and low-background fluorescent sensor platform is developed to detect nucleic acids based on the combination of δ-FeOOH nanosheets with Exo III–assisted target-recycling signal amplification. δ-FeOOH nanosheets, as the quenchers, are conjugated with the dye-labeled ssDNA probes. The dye-labeled ssDNA probes integrate with the DNA targets to form a double-strand DNA complex (dsDNA). Then, the dye-labeled ssDNA probes in the dsDNA complex will be gradually hydrolyzed into short fragments by Exo III, and the fluorescence signal is recovered due to the weaker bind affinity between short fragments and δ-FeOOH nanosheets (Wu et al., 2020). Markedly, the most suitable environment should be provided for boosting Exo III activity, and this sensing platform would become a universal approach for optimizing the early detection of DNA mutation.

### Nucleic Acid Probe–Based Fluorescent Sensing Systems in Cancer Treatment

Abnormal changes in tumor suppressor genes, oncogenes, and other molecules are found in various cancers. Thus, precisely targeting these aberrant molecules via nucleic acid probe–based fluorescent sensing is prospective for guiding and optimizing cancer gene–based individual treatment. There are several noble fluorescence sensing strategies, including multifunctional primer–integrated MBs (MP-MBs), ultrasensitive rolling circle amplification (THP-RCA), increasingly branched rolling circle amplification (IB-RCA), and polymethylmethacrylate nanoparticle (PMMA-NP)–modified MBs (Table 1).

p53 is an essential tumor suppressor, and targeting p53 mutation should also be concerned (D'Orazi et al., 2021). Presently, the MP-MB probe has been developed to detect p53 gene. Compared with the traditional MBs, MP-MBs can not only selectively identify the targets and sensitively transmit a hybridization signal but also act as the primer during enzymatic polymerization. Specifically, hybridization of MP-MBs with p53 gene can restore the fluorescence intensity and provoke the pre-locked primer by changing the molecular configuration of MP-MBs, further targeting p53 mutation and instructing p53 gene–guided individual therapy (Xu et al., 2015). MP-MBs do not require any chemical modification, and with less species requirement, they have wider sequence diversity and preserved intrinsic bioactivity.

STAT3 is a potent proto-oncogene, and screening STAT3 gene is useful for cancer therapy (Kryczek et al., 2014). A novel THP-RCA strategy is designed to ultrasensitively detect human proto-oncogenes *via* conjugating with target-catalyzed hairpin structure–mediated padlock cyclization. For the system, hairpin probe (HP) 1 is formed as the cyclization template and RCA reaction primer and HP2 is the padlock probe. The two probes fold into a hairpin structure via self-hybridization. In the presence of STAT3 DNA, HP2 hybridizes with HP1 in an end-to-end manner. Then, HP2 is cyclized by ligase on the HP1 template; the cyclized HP2 enables the RCA and generates a long tandem ssDNA product that is capable of hybridizing with considerable quantity of MBs. Subsequently, the amplified fluorescence value represents the ultrasensitive detection of STAT3 gene (Song et al., 2019). Moreover, the sensing system is suitable for target detection in human serum.

Similarly, IB-RCA is constructed for highly sensitively detecting and targeting the colorectal cancer (CRC) gene, Kras gene codon 12, which comprises a padlock probe (PP) and an MB (Li et al., 2016). The PP is circularized after hybridization with the DNA target, while the stem of the MB is opened by the DNA target. The newly opened MB hybridizes with the circularized PP to generate a long tandem ssDNA product, consequently triggering the next RCA reactions and producing a dramatically amplified fluorescence signal (Li et al., 2016). It is worth noting that IB-RCA efficiently transduces the fluorescence signal in a simpler way compared with conventional amplification methods.

In addition, targeting mRNAs is the other cardinal avenue to cancer treatment. Survivin is an overexpressed anti-apoptotic protein and considered a pharmacological target for effective anticancer therapy (Meiners et al., 2021). Survivin MBs can selectively detect survivin mRNA through embedding into the cells with the assistance of Lipofectamine, but MBs might be degraded by enzymes *in vivo* (Bishop et al., 2015). In order to overcome this problem, biocompatible core–shell PMMA-NPs serve as the carrier of MBs to specifically target survivin mRNA in A549 human lung adenocarcinoma epithelial cells, which suppresses cancer cell proliferation (Adinolfi et al., 2017). PMMA-NPs consist of a fluorescein-modified hydrophobic PMMA core and an external hydrophilic shell functionalized with primary amine groups and quaternary ammonium salts. Interestingly, the PMMA-NP carrier has higher biocompatibility, lower cytotoxicity to healthy cells, higher biological inertness, lower synthesis costs, and higher selectivity, as well as prolonging the drug half-life in the human body compared with classical transfection reagents such as Lipofectamine, which extend the application of PMMA-NPs (Brandts et al., 2021).

## The Application of Nucleic Acid Probe–Based Fluorescent Imaging in Cancer Diagnosis and Treatment

**FIGURE 2:**
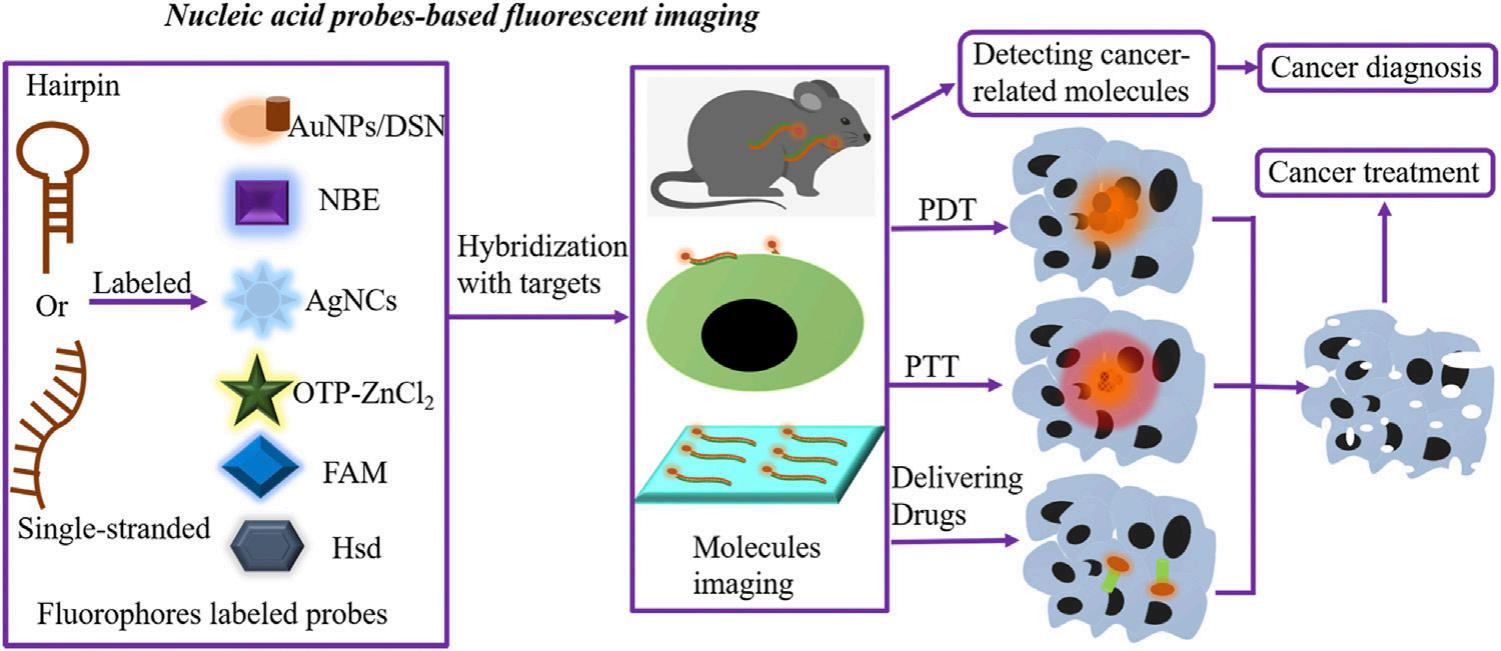
Nucleic acid probe–based fluorescent imaging. Hairpin or single-stranded nucleic acid probes are labeled with fluorophores to form nucleic acid probe–based fluorescent imaging platforms. Novel fluorophores include AuNPs/DSN, AgNC-MBs, FAM, OTP-ZnCl_2_, Hsd, and NBE. In the presence of targets, fluorophore-labeled probes hybridize targets, further performing molecular imaging and locating molecules expressed on the surface of cells or tissues and targeting cancer cells in living samples. The fluorescent imaging platforms can detect cancer-related molecules, resulting in an elevated efficiency of cancer diagnosis. Meanwhile, these imaging methods are utilized for delivering anticancer drugs and guiding PDT and PTT, further killing cancer cells by *in situ* imaging of low-abundance biomarkers. AgNCs, silver nanoclusters; AuNPs, gold nanoparticles; MBs, molecular beacons; DSN, double-specific nuclease; PDT, photodynamic therapy; PTT, photothermal therapy.

At present, even if nucleic acid probe–based fluorescent sensing has made significant progress in cancer diagnosis and therapy, it cannot detect the cancer-relevant targets *in situ*. It is worth noting that nucleic acid probe–based fluorescent imaging can visualize the cancer target expression, composed of visualizing the changes in molecule conformation, locating surface molecules, and targeting cancer cells in living samples with a high spatiotemporal resolution, resulting in an elevated efficiency of cancer diagnosis and treatment (Figure 2). Due to the excellent functions of RNA during cancer development, the investigation of multi-fluorophore color RNA probes is required for understanding the correlation of gene expression and interaction between nucleic acids (Okamoto, 2011).

### Nucleic Acid Probe–Based Fluorescent Imaging Platforms in Cancer Diagnosis

miRNAs have become ideal and noninvasive cancer biomarkers. To accomplish better and faster miRNA imaging, Au nanoparticles (AuNPs)/double-specific nuclease (DSN), AgNC-generating MBs (AgNC-MBs), reduced graphene oxide (rGO), FAM, OTP-ZnCl_2_, Hsd, and NBE-modified fluorescent probes are applied for fabricating imaging platforms and measuring mutant-type targets in diagnosis of various cancers (Figure 2; Table 2).

**TABLE 2:** Application of nucleic acid probe–based fluorescent imaging in cancer diagnosis and treatment.

Fluorogenic imaging	Probes	Cancers	Targets	Application	Ref.
AuNPs/DSN@CM	ssDNA	Breast cancer	Multiplex miRNAs	Diagnosis	Lu et al. (2020)
AgNC-MBs	ssDNA	Breast cancer	miRNA-21 and let-7a	Diagnosis	Peng et al. (2019b)
rGO	ssDNA	Breast cancer	miRNA-451a and miRNA-214-3p	Diagnosis	Xiong et al. (2021)
OTP-ZnCl_2_	RNA	HCC	Total RNA	Diagnosis	Wang et al. (2016)
Hsd	RNA	Cervical carcinoma	Total RNA	Diagnosis	Li et al. (2013)
NEB	RNA	Breast cancer and HCC	Total RNA	Diagnosis	Yao et al. (2018)
g-C3N4 nanosheet	ssDNA	Lung cancer	Survivin mRNA	PDT	Xiang et al. (2020)
AgNCs	ssDNA	Various cancers	Glycans	PTT	Wu et al. (2018)
rGONS	ssDNA	Various cancers	p53 and p21 mRNA	Treatment	Fan et al. (2019)
AuNP–MB–Dox	ssDNA	Breast cancer	Cyclin D1 mRNA	Treatment	Qiao et al. (2011)

AgNCs, silver nanoclusters; AuNPs, gold nanoparticles; CM, cell membrane; DSN, double-specific nuclease; g-C3N4, graphitic carbon nitride; HCC, hepatocellular carcinoma; MBs, molecular beacons; PDT, photodynamic therapy; PTT, photothermal therapy; rGO, reduced graphene oxide; rGONS, reduced graphene oxide nanosheet; ssDNA, single-stranded DNA.

Highly efficient cellular transfection and intracellular signal amplification are basis for low-abundance miRNA imaging (Chen et al., 2019a; Lu et al., 2020). A study uses AuNPs/DSN to encapsulate the functional cancer cell membrane (CM) vesicle, and AuNPs are modified with three types of fluorescent probes. The AuNPs/DSN@CM can specifically target the cancer cell, and the internalized AuNPs/DSN@CM further recognizes the miRNA targets and induces DSN-based recycle signal amplification, leading to simultaneous detection of multiple miRNAs. This approach has successfully analyzed and monitored the dynamic changes in oncogenic miRNAs in breast cancer cells with high sensitivity (Lu et al., 2020). Compared with traditional AuNPs, AuNPs/DSN@CM exhibits the higher transfection efficiency, biocompatibility, and specificity.

Favorably, the fluorescent AgNC-MBs are economical alternatives for detecting multiple nucleic acids (Del Bonis-O'Donnell et al., 2016; Huang et al., 2018). However, most of AgNC-MBs have limited versatility; the reason is that fluorescence properties of DNA-AgNCs will be severely damaged when the AgNC-stabilizing sequence is embedded into the MB sequence. Based on toehold-mediated DNA strand displacement, a new type of AgNC-MB is constructed by combining with total internal reflection fluorescence (TIRF)–based single-molecule fluorescence imaging (Peng et al., 2019b). The AgNC-MB platform can simultaneously measure two breast cancer–related miRNAs (miRNA-21 and let-7a) and distinguish the mutant-type targets at low abundance (Peng et al., 2019a). In addition, miRNA-451a and miRNA-214-3p are meaningful biomarkers for breast cancer; the novel rGO-modified DNA nanoprobe is prepared for simultaneous dual-color imaging of miRNA-451a and miRNA-214-3p (Xiong et al., 2021). Above all, the AgNC-MBs and rGO-modified imaging platforms provide versatile methods for sensitively and simultaneously imaging multiple miRNA biomarkers. Notably, AgNCs have been employed in single-molecule microscopy, molecular logic devices, and metal ion sensing (Adinolfi et al., 2017).

Currently, fluorescent RNA probes are developing. The OTP-ZnCl_2_ complex has a better interaction with nucleolus RNA than DNA, and it can stably insert into the inside of RNA based on the hydrogen bonds between OTP-ZnCl_2_ and RNA, between the oxime group and the base pair of RNA (Wang et al., 2016). Because of the outstanding cell permeability, low cytotoxicity, and counterstain compatibility, OTP-ZnCl_2_ has become a favorable dye for designing selective RNA fluorescent probes and two-photon fluorescence imaging. In this study, OTP-ZnCl_2_–based fluorescent RNA probes are allowed for accurate RNA imaging within hepatocellular carcinoma (HCC) cells (Wang et al., 2016). In addition, the near-infrared and cell-impermeant fluorescent dye Hsd is utilized to modify RNA probes owing to its selective response to RNA, and it can enter into the living cells for selective RNA staining and imaging with low cytotoxicity and fluorescence quantum yield. But the process needs the assistance of cucurbit[7]uril (CB7) to strengthen the potential of Hsd in cancer diagnosis (Li et al., 2013). Besides, NBE, an NIR fluorescent probe, has no response to DNA. NBE-modified RNA probes are utilized for fulfilling excellent RNA imaging in live breast cancer and HCC cells with good photostability, high selectivity, and fast response to RNA, which facilitates the diagnosis of various cancers according to RNA contents (Yao et al., 2018).

### Nucleic Acid Probe–Based Fluorescent Imaging Systems in Cancer Treatment

Likewise, nucleic acid probe–based fluorescent imaging is an available approach for guiding cancer treatment and improving the therapeutic efficacy with *in situ* imaging of low-abundance nucleic acid targets. Currently, some imaging methods are utilized for delivering anticancer drugs and guiding photodynamic therapy (PDT) and photothermal therapy (PTT) (Figure 2; Table 2).

Recently, the water-dispersible graphitic carbon nitride (g-C3N4) nanosheet has been considered an excellent nanocarrier functioned with CHA amplification, and it is applied for self-tracking transfection of DNA hairpin probes (Xiang et al., 2020; Lin et al., 2021a). The cancer-related mRNAs will efficiently initiate the DNA hairpin probes, ultimately leading to an amplified fluorescence signal *via* hybridization and mRNA displacement. Then, the enhanced fluorescence imaging will sensitively analyze the low-abundance cancer-relevant mRNAs, directly track the location, and guide precise PDT of cancers upon light irradiation (Xiang et al., 2020). Presently, the g-C3N4 nanosheet–based nanoassembly has been used for low-abundance survivin mRNA imaging and anticancer PDT, which do not show obvious side effects (Xiang et al., 2020).

As we all know, aberrant alterations of glycans are involved in many types of cancers. Herein, DNA-stabilized AgNC probes have been presented for label-free fluorescence imaging of cell surface glycans and fluorescence-guided PTT. In this pattern, surface glycans are specifically labeled by DNA-AgNC fluorescent probes *via* the dibenzocyclooctyne (DBCO)-functioned and DNA-initiated hybridization chain reaction (HCR). Then, DNA-AgNC probes produce the amplified signal, subsequently killing cancer cells and inhibiting cancer growth due to the remarkable photothermal properties of the HCR. Furthermore, DNA-AgNCs can dramatically reduce the cost and the instability of fluorescent dyes, and the HCR prevents the introduction of excessive azido-sugars and ensures apparent fluorescence. These results present the high value of the fluorescence imaging nanoplatform in visualizing specific glycans and guiding anticancer PTT (Wu et al., 2018).

The p53 and p21 genes play vital roles in blocking cancer development; it is important to monitor mRNA levels of the two markers (Lei et al., 2020). Herein, a reduced graphene oxide nanosheet (rGONS)–modified nanosystem is constructed for *in situ* and real-time p53 and p21 mRNA imaging by adsorbing the FAM-labeled p21 probe (P21) and Cy5-labeled p53 probe (P53). Once the two fluorescence probes hybridize with corresponding targets, the formation of DNA/RNA duplexes directly facilitates the release of probes from the rGONS surface and then restores the fluorescence signal (Fan et al., 2019). Therefore, the nanosystem *in situ* reveals a p53 and p21 mRNA–related regulatory process, which is practicable for drug screening and therapy evaluation in clinics.

Doxorubicin (Dox) is a common anticancer drug, and fluorophore-modified Dox is essential for cancer targeted therapy by intercalation within DNA/RNA. Herein, Dox carriers directly impact the therapeutic efficiency, and MB-functionalized AuNPs are identified as superior carriers to deliver fluorescent Dox. When MBs selectively interact with mRNA targets, fluorescent Dox is released from the AuNP–MB–Dox complex. The released Dox is positively correlated with the quantities of mRNA targets (Qiao et al., 2011). This strategy selectively detects the concentration of cyclin D1 mRNA in breast cancer and induces cyclin D1 + breast cancer cell apoptosis. Obviously, AuNP-MBs are the ideal carriers for transporting anticancer drugs, as they can specifically interact with cancer-relevant mRNA targets and kill cancer cells with lower side effects (Qiao et al., 2011).

## Discussion

All in all, the applications of fluorescent biosensors and imaging technologies are increasingly widespread. However, there are some defects that need to be improved: i) The nucleic acid probes might lose the ability to hybridize with target strands when the target sequences form secondary structures such as hairpins or quadruplexes, subsequently disturbing the surrounding sequences (Ming et al., 2019). Thus, an open strand–based model is required for eliminating the influence of complicated secondary structures. The model would be conducted for observing low-abundance DNA mutations in cancer samples, further improving cancer gene–based individual therapy. ii) We should also focus on the development of other fluorescence signaling techniques, such as lifetime, correlation spectroscopy, polarization, and localization; they are excellent carriers for delivering information and exploring molecular interactions and DNA structures, which will make significant advancements in cancer diagnostics and theranostics (Su et al., 2012; Adinolfi et al., 2017).

Some nucleic acid probe–based fluorescent sensing platforms are simultaneously labeled with fluorogen and quencher. The synthesis of both fluorogen and quencher is complex, and the relative distance between fluorogen and quencher is difficult to control, which may lead to false-positive and false-negative results (Ou et al., 2017). In order to realize more specific molecular recognition and more accurate quantification of target molecules, studies are supposed to focus on designing new nucleic acid probes with more chemical functionalities and less nonspecific interactions. Even though fluorescent probe–labeled nucleic acid aptamers exert brilliant functions with low cytotoxicity and high specificity, the performance of aptamer-based sensors remains to be improved due to fast nuclease degradation, rapid renal excretion, and weaker binding affinity (Tan et al., 2019).

Nucleic acid fluorescent probe–based imaging technology has attracted widespread attention, which can display quantitative maps according to the concentrations of target molecules in living samples. Although numerous nucleic acid probes have sufficient sensitivity and selectivity for *in vitro* imaging of various targets, there are several scientific and technical challenges to *in situ* and *in vivo* fluorescence imaging: i) the complexity of tumor microenvironment (TME) might cause damage to normal cells and non-target molecules (Lin et al., 2021b) and ii) the high background of enzymatic catalysis dramatically decreases the feasibility of *in vivo* fluorescence imaging (Ferrero et al., 2021). Thus, novel fluorophores with high quantum yield need to be explored for eliminating the background signal and reducing the perturbation to normal biological processes, which is vital for monitoring the enzymatic processes with greater temporal and spatial resolution. Currently, a number of aptamer-based methods for *in vivo* fluorescence imaging have been reported, such as fluorescent dyes, QDs, or upconversion nanoparticle–labeled aptamers (Bagalkot et al., 2007; Kim et al., 2012).

Although nucleic acid probe–based fluorescent sensing and imaging systems have made some progress, several drawbacks need to be ameliorated, including low sample throughput, defective reproducibility, insufficient quantitation accuracy, high operation costs, complicated procedures, and long assay period (Fang et al., 2019). To solve these deficiencies, miniaturized and automated nanodevices would be rapidly developed *via* integrating fluorescence-labeled nucleic acid probes with high-throughput technologies, such as lateral flow devices, microfluidic chips, or microarray chips (Fang et al., 2019). These creative nanodevices would achieve tremendous advancements in cancer diagnostics and theranostics through real-time monitoring of biological processes, rapidly identifying targets and characterizing enzymes in a complex system (Fang et al., 2019). Nevertheless, how to assist them to exert more sophisticated functions in complicated biological environments remains to be explored.

## Abbreviation:

AgNCs, silver nanoclusters; AuNPs, gold nanoparticles; CDs, carbon dots; CM, cell membrane; CRC, colorectal cancer; Cyt c, cytochrome c; DSN, double-specific nuclease; FCM, flow cytometry; g-C3N4, graphitic carbon nitride; HCC, hepatocellular carcinoma; IB-RCA, increasingly branched rolling circle amplification; MBs, molecular beacons; MP, multifunctional primer; PDT, photodynamic therapy; PMMA-NPs, polymethylmethacrylate nanoparticles; PNA, peptide nucleic acid; PTT, photothermal therapy; rGO, reduced graphene oxide; rGONS, reduced graphene oxide nanosheet; ssDNA, single-stranded DNA; THP-RCA, ultrasensitive rolling circle amplification; TME, tumor microenvironment.
